# Ischemia‐modified albumin in rheumatic diseases: A systematic review and meta‐analysis

**DOI:** 10.1002/iid3.1324

**Published:** 2024-06-18

**Authors:** Arduino A. Mangoni, Angelo Zinellu

**Affiliations:** ^1^ Discipline of Clinical Pharmacology, College of Medicine and Public Health Flinders University Adelaide Australia; ^2^ Department of Clinical Pharmacology Flinders Medical Centre, Southern Adelaide Local Health Network Adelaide Australia; ^3^ Department of Biomedical Sciences University of Sassari Sassari Italy

**Keywords:** acidosis, biomarkers, IMA, ischemia, ischemia‐modified albumin, oxidative stress, rheumatic diseases

## Abstract

**Introduction:**

The identification of novel, easily measurable disease biomarkers might enhance the diagnosis and management of patients with rheumatic diseases (RDs). We conducted a systematic review and meta‐analysis of ischemia‐modified albumin (IMA), a marker of oxidative stress, acidosis, and ischemia, in RD patients and healthy controls.

**Methods:**

We searched PubMed, Web of Science, and Scopus from inception to January 15, 2024. The risk of bias and the certainty of evidence were assessed using the Joanna Briggs Institute Critical Appraisal Checklist and GRADE, respectively.

**Results:**

In 20 studies investigating a total of 1188 RD patients (mean age 45 years, 64% females) and 981 healthy controls (mean age 44 years, 66% females), RD patients had significantly higher IMA concentrations when compared to controls (standard mean difference, SMD = 0.50, 95% CI: 0.18−0.83, *p* = .003; *I* ^2^ = 92.4%, *p* < .001; low certainty of evidence). In subgroup analysis, the pooled SMD was significantly different in studies investigating ankylosing spondylitis (*p* < .001), Behçet's disease (*p* < .001), and rheumatoid arthritis (*p* = .033), but not familial Mediterranean fever (*p* = .48). Further associations were observed between the pooled SMD and the broad classification of autoimmune and/or autoinflammatory diseases, the study country, and the method used to measure IMA.

**Conclusion:**

Our study suggests that IMA is a promising biomarker of oxidative stress, acidosis, and ischemia, as it can effectively discriminate between patients with different types of RDs and healthy controls. Our results warrant confirmation in longitudinal studies of patients with different types of RDs and different ethnicities (PROSPERO registration number: CRD42024509126).

## Graphical abstract

Our study suggests that ischemia‐modified albumin is a promising biomarker of oxidative stress, acidosis, and ischemia in different types of rheumatic diseases. Our results warrant confirmation in longitudinal studies of patients with different types of rheumatic diseases and different ethnicity.

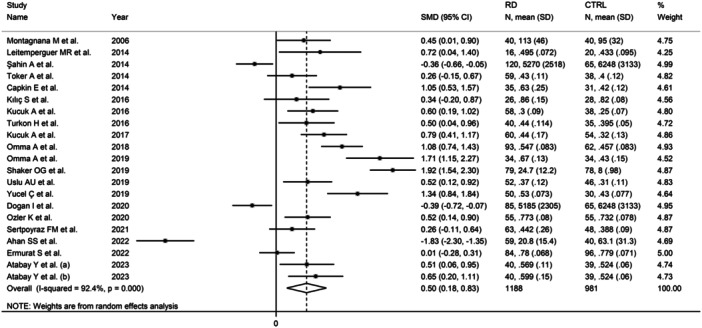

## 1 INTRODUCTION

The terminology “rheumatic diseases (RDs)” includes a significant number of chronic and disabling conditions characterized by excess inflammation and oxidant stress involving the musculoskeletal system as well as other organs and systems. By and large, RDs may have a predominantly autoimmune (e.g., progressive systemic sclerosis, pSS, rheumatoid arthritis, RA, systemic lupus erythematosus, SLE, Sjogren's syndrome, SS, systemic sclerosis, SSc), a mixed‐autoimmune‐autoinflammatory (e.g., ankylosing spondylitis, AS, axial spondyloarthritis, axSpA, psoriatic arthritis, PsA, and Behçet's disease, BD), or an autoinflammatory component (e.g., familial Mediterranean fever, FMF).^1^, ^2^, ^3^ Given the existence of different classifications of RDs that have also evolved over time, studies investigating the epidemiology of RDs at the population level have generally provided inconsistent results regarding their prevalence in various geographical locations.^4^, ^5^, ^6^, ^7^, ^8^, ^9^ This issue notwithstanding, it is well accepted that individual RDs impose a significant burden on the community and healthcare systems worldwide despite significant advances in pharmacological and nonpharmacological treatments.^10^, ^11^, ^12^, ^13^, ^14^, ^15^, ^16^, ^17^, ^18^, ^19^, ^20^, ^21^, ^22^

One active area of research in RDs involves the capacity to better identify early forms of the disease to rapidly implement treatments targeting the dysregulated immune and inflammatory pathways and, potentially, improve long‐term clinical outcomes.^23^, ^24^, ^25^, ^26^, ^27^, ^28^ Therefore, significant efforts have been made to discover novel biomarkers of RDs that facilitate early diagnosis and complement the information provided by clinical assessment and conventional inflammatory markers, for example, C‐reactive protein (CRP) and erythrocyte sedimentation rate (ESR).^29^, ^30^, ^31^, ^32^, ^33^, ^34^, ^35^, ^36^, ^37^ One candidate biomarker that has been increasingly studied in RDs over the last 15 years is ischemia‐modified albumin (IMA). IMA is generated from chemical modifications targeting the N‐terminal sequence of native albumin in the presence of ischemic processes.^38^ Such chemical modifications can also be triggered by oxidative stress and acidosis.^38^ Alterations in the concentrations of IMA have been investigated in physiological processes, for example, physical exercise and pregnancy,^39^, ^40^ as well as cerebrovascular disease, ischemic heart disease, and heart failure,^41^, ^42^, ^43^, ^44^, ^45^, ^46^, ^47^ neurological disorders,^48^, ^49^ diabetes mellitus,^50^ and cancer.^51^, ^52^ Given that the formation of IMA reflects, in addition to ischemia, the presence of oxidative stress and acidosis, commonly observed in patients with RDs,^53^, ^54^, ^55^, ^56^, ^57^, ^58^, ^59^, ^60^ we assessed the potential role of IMA as a biomarker by conducting a systematic review and meta‐analysis of circulating IMA concentrations in RD patients and healthy controls. We speculated that RD patients had significantly higher IMA concentrations compared to healthy controls, highlighting the potential diagnostic role of IMA in RDs.

## 2 MATERIALS AND METHODS

### 2.1 Search strategy, inclusion criteria, and study selection

We systematically searched the electronic databases PubMed, Web of Science, and Scopus from inception to January 15, 2024, using the following terms: “IMA” OR “ischemia modified albumin” OR “ischemia‐modified albumin” AND “rheumatic diseases” OR “rheumatoid arthritis” OR “psoriatic arthritis” OR “reactive arthritis” OR “ankylosing spondylitis” OR “systemic lupus erythematosus” OR “systemic sclerosis” OR “scleroderma” OR “Sjogren's syndrome” OR “connective tissue diseases” OR “vasculitis” OR “Behçet's disease” OR “idiopathic inflammatory myositis” OR “polymyositis” OR “dermatomyositis” OR “gout” OR “pseudogout” OR “systemic vasculitis” OR “ANCA‐associated vasculitis” OR “Takayasu's arteritis” OR “polyarteritis nodosa” OR “osteoarthritis” OR “fibromyalgia” OR “granulomatous polyangiitis” OR “Henoch‐Schonlein purpura” OR “Wegener's granulomatosis” OR “familial Mediterranean fever”. Two independent investigators screened each abstract and, if potentially relevant, the full‐text article according to the following inclusion criteria: (i) measurement of IMA in plasma or serum, (ii) comparison between RD patients and healthy controls using a case‐control design, (iii) age ≥18 years, (iv) use of English language, and (v) availability of the full‐text of the article. The references of individual articles were hand‐searched for additional studies.

The following variables were independently extracted from each article and transferred to an electronic spreadsheet for further analysis: year of publication, first author, type of RD, the country where the study was conducted, number of participants, age, male‐to‐female ratio, body mass index (BMI), CRP, ESR, mean RD duration, and analytical method used to measure IMA.

We used the Joanna Briggs Institute Critical Appraisal Checklist for analytical studies to assess the risk of bias,^61^ and the Grades of Recommendation, Assessment, Development and Evaluation (GRADE) Working Group system to assess the certainty of evidence.^62^ We complied with the Preferred Reporting Items for Systematic Reviews and Meta‐Analyses (PRISMA) 2020 statement (Tables S1 and S2),^63^ and registered the study protocol in an international register (PROSPERO registration number: CRD42024509126).

### 2.2 Statistical analysis

We calculated standardized mean differences (SMDs) and 95% confidence intervals (CIs) to generate forest plots of continuous data and to assess possible differences in IMA concentrations between RD patients and healthy controls (statistical significance was set at a *p*‐value < .05). If necessary, means and standard deviations were calculated from medians and interquartile ranges or from medians and ranges using published methods^64^ or the Graph Data Extractor Software. Heterogeneity of the SMD across studies was investigated using the *Q* statistic (significance level at *p* < .10), and a random‐effect model based on the inverse‐variance method was used in the presence of high heterogeneity.^65^, ^66^ Sensitivity analysis was performed to confirm the stability of the results of the meta‐analysis.^67^ Publication bias was investigated using the Begg's adjusted rank correlation test and the Egger's regression asymmetry test (statistical significance was set at a *p*‐value < .05).^68^, ^69^ We performed univariate meta‐regression and subgroup analyses to assess possible associations between the effect size and year of publication, type of RD, study country, sample size, age, male‐to‐female ratio, BMI, CRP, ESR, mean RD duration, and method used to measure IMA. Statistical analyses were performed using Stata 14 (Stata Corp.).

## 3 RESULTS

### 3.1 Systematic search and study selection

**Figure 1:**
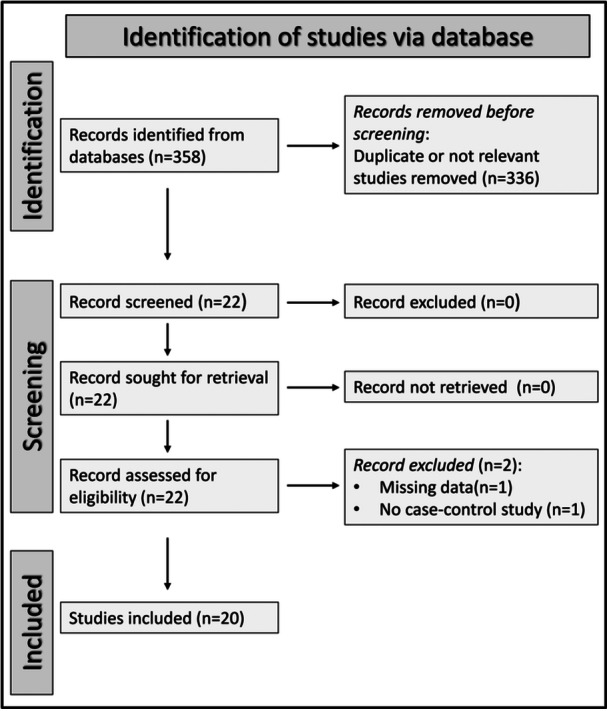
PRISMA 2020 flow diagram.

A flow chart of the screening process is described in Figure 1. After initially identifying 358 articles, we excluded 336 because they were either duplicates or irrelevant. After reviewing the full text of the remaining 22 articles, a further two were excluded because of missing data (one study) and no case‐control design (one study). Therefore, 20 studies were selected for analysis^70^, ^71^, ^72^, ^73^, ^74^, ^75^, ^76^, ^77^, ^78^, ^79^, ^80^, ^81^, ^82^, ^83^, ^84^, ^85^, ^86^, ^87^, ^88^, ^89^ (Table 1). The risk of bias was low in 18 studies,^70^, ^71^, ^72^, ^73^, ^76^, ^77^, ^78^, ^79^, ^80^, ^81^, ^82^, ^83^, ^84^, ^85^, ^86^, ^87^, ^88^, ^89^ moderate in one,^75^ and high in the remaining one^74^ (Table 2). The cross‐sectional nature of the selected studies primarily accounted for the low initial certainty of evidence (level 2).

**Table 1:** Characteristics of the studies investigating ischemia‐modified albumin in patients with rheumatic diseases and healthy controls.

Study	Healthy controls				Patients with rheumatic diseases				Type of rheumatic disease
	*n*	Age (years)	M/F	IMA (mean ± SD)	*n*	Age (years)	M/F	IMA (mean ± SD)	
Montagnana M et al. 2006, Italy^70^	40	55	0/40	95 ± 32	40	56	0/40	113 ± 46	SSc
Leitemperguer MR et al. 2014, Brazil^71^	20	51	5/15	0.433 ± 0.095	16	57.7	5/11	0.495 ± 0.072	RA
Şahin A et al. 2014, Turkey^72^	65	NR	22/43	6248 ± 3133	120	NR	45/75	5270 ± 2518	FMF
Toker A et al. 2014, Turkey^73^	38	42	4/34	0.4 ± 0.12	59	44	6/53	0.43 ± 0.11	Fibromyalgia
Capkin E et al. 2014, Turkey^74^	31	38	NR	0.42 ± 0.12	35	38.1	NR	0.63 ± 0.25	BD
Kılıç S et al. 2016, Turkey^75^	28	NR	NR	0.82 ± 0.08	26	40.3	NR	0.86 ± 0.15	BD
Kucuk A et al. 2016, Turkey^76^	38	41	21/17	0.25 ± 0.07	58	37	28/30	0.30 ± 0.09	FMF
Turkon H et al. 2016, Turkey^77^	35	36	24/11	0.395 ± 0.05	40	39	27/13	0.44 ± 0.114	AS
Kucuk A et al. 2017, Turkey^78^	54	42	32/22	0.32 ± 0.13	60	42	45/15	0.44 ± 0.17	AS
Omma A et al. 2018, Turkey^79^	62	Matched	Matched	0.457 ± 0.083	93	38	57/36	0.547 ± 0.083	BD
Omma A et al. 2019, Turkey^80^	34	46	23/11	0.43 ± 0.15	34	45	24/10	0.67 ± 0.13	IgAV
Shaker OG et al. 2019, Egypt^81^	78	39	16/62	8.00 ± 0.98	79	38	10/69	24.7 ± 12.2	RA
Uslu AU et al. 2019, Turkey^82^	46	44	18/28	0.31 ± 0.11	52	47	11/41	0.37 ± 0.12	RA
Yucel Ç et al. 2019, Turkey^85^	30	42	18/12	0.43 ± 0.077	50	39	33/17	0.53 ± 0.073	BD
Dogan I et al. 2020, Turkey^83^	65	54	22/43	6248 ± 3133	85	55	9/76	5185 ± 2305	SS
Ozler K et al. 2020, Turkey^84^	55	59	Matched	0.732 ± 0.078	55	61	Matched	0.773 ± 0.08	OA
Sertpoyraz FM et al. 2021, Turkey^86^	48	40	26/22	0.388 ± 0.09	63	44	35/28	0.442 ± 0.26	AS
Ahn SS et al. 2022, South Korea^87^	40	NR	NR	63.1 ± 31.3	59	64	21/38	20.81 ± 15.44	AAV
Ermurat S et al. 2022, Turkey^88^	96	37	9/87	0.779 ± 0.071	84	39	4/80	0.78 ± 0.068	SLE
Atabay Y et al. (a) 2023, Turkey^89^	39	41	10/29	0.524 ± 0.06	40	34	14/26	0.569 ± 0.11	FMF
Atabay Y et al. (b) 2023, Turkey^89^	39	41	10/29	0.524 ± 0.06	40	38	17/23	0.599 ± 0.15	AS

*Note*: IMA values are reported as absorbance units or U/mL.

Abbreviations: AAV, ANCA‐associated vasculitis; AS, ankylosing spondylitis; BD, Behcet disease; FMF, familial Mediterranean fever; IgAV, immunoglobulin A vasculitis; IMA, ischemia‐modified albumin; M/F, male to female ratio; NR, not reported; OA, osteoarthritis; RA, rheumatoid arthritis; SLE, systemic lupus erythematosus; SpA, spondylarthritis; SS, Sjögren's syndrome; SSc, systemic sclerosis.

**Table 2:** Assessment of the risk of bias using the Joanna Briggs Institute critical appraisal checklist.

Study	Were the inclusion criteria clearly defined?	Were the subjects and the setting described in detail?	Was the exposure measured in a reliable way?	Were standard criteria used to assess the condition?	Were confounding factors identified?	Were strategies to deal with confounding factors stated?	Were the outcomes measured in a reliable way?	Was appropriate statistical analysis used?	Risk of bias
Montagnana et al.^70^	Yes	Yes	Yes	Yes	No	No	Yes	Yes	Low
Leitemperguer et al.^71^	Yes	Yes	Yes	Yes	No	No	Yes	Yes	Low
Şahin et al.^72^	Yes	Yes	Yes	Yes	No	No	Yes	Yes	Low
Toker et al.^73^	Yes	Yes	Yes	Yes	No	No	Yes	Yes	Low
Capkin et al.^74^	No	No	Yes	No	No	No	Yes	Yes	High
Kılıç et al.^75^	Yes	No	Yes	Yes	No	No	Yes	Yes	Moderate
Kucuk et al.^76^	Yes	Yes	Yes	Yes	No	No	Yes	Yes	Low
Turkon et al.^77^	Yes	Yes	Yes	Yes	No	No	Yes	Yes	Low
Kucuk et al.^78^	Yes	Yes	Yes	Yes	No	No	Yes	Yes	Low
Omma et al.^79^	Yes	Yes	Yes	Yes	No	No	Yes	Yes	Low
Omma et al.^80^	Yes	Yes	Yes	Yes	No	No	Yes	Yes	Low
Shaker et al.^81^	Yes	Yes	Yes	Yes	No	No	Yes	Yes	Low
Uslu et al.^82^	Yes	Yes	Yes	Yes	No	No	Yes	Yes	Low
Yucel et al.^85^	Yes	Yes	Yes	Yes	No	No	Yes	Yes	Low
Dogan et al.^83^	Yes	Yes	Yes	Yes	No	No	Yes	Yes	Low
Ozler et al.^84^	Yes	Yes	Yes	Yes	Yes	Yes	Yes	Yes	Low
Sertpoyraz et al.^86^	Yes	Yes	Yes	Yes	No	No	Yes	Yes	Low
Ahn et al.^87^	Yes	Yes	Yes	Yes	Yes	Yes	Yes	Yes	Low
Ermurat et al.^88^	Yes	Yes	Yes	Yes	No	No	Yes	Yes	Low
Atabay et al.^89^	Yes	Yes	Yes	Yes	Yes	Yes	Yes	Yes	Low

### 3.2 Study characteristics

Twenty studies, including 21 group comparators, investigated IMA in a total of 1188 RD patients (mean age 45 years, 64% females) and 981 healthy controls (mean age 44 years, 66% females)^70^, ^71^, ^72^, ^73^, ^74^, ^75^, ^76^, ^77^, ^78^, ^79^, ^80^, ^81^, ^82^, ^83^, ^84^, ^85^, ^86^, ^87^, ^88^, ^89^ (Table 1). Sixteen studies were conducted in Turkey,^72^, ^73^, ^74^, ^75^, ^76^, ^77^, ^78^, ^79^, ^80^, ^82^, ^83^, ^84^, ^85^, ^86^, ^88^, ^89^ one in Italy,^70^ one in Brazil,^71^ one in Egypt,^81^ and one in South Korea.^87^ Four study groups included patients with AS,^77^, ^78^, ^86^, ^89^ four with BD,^74^, ^75^, ^79^, ^85^ three with RA,^71^, ^81^, ^82^ three with FMF,^72^, ^76^, ^89^ one with SSc,^70^ one with fibromyalgia,^73^ one with IgA vasculitis (IgAV),^80^ one with SS,^83^ one with osteoarthritis (OA),^84^ one with ANCA‐associated vasculitis (AAV),^87^ and one with SLE.^88^ IMA was measured using the albumin cobalt binding test (ACB) test in 14 group comparators,^73^, ^75^, ^76^, ^77^, ^78^, ^79^, ^80^, ^82^, ^84^, ^85^, ^86^, ^88^, ^89^ an enzyme‐linked immunosorbent assay (ELISA) in two,^81^, ^87^ an automatic analyzer in two,^70^, ^71^ whereas the remaining three did not report the analytical method used.^72^, ^74^, ^83^ Disease duration, reported in 11 study groups, ranged between 1.7 and 9 years.^70^, ^73^, ^75^, ^78^, ^79^, ^81^, ^82^, ^83^, ^85^, ^87^, ^88^

### 3.3 Results of the meta‐analysis

**Figure 2:**
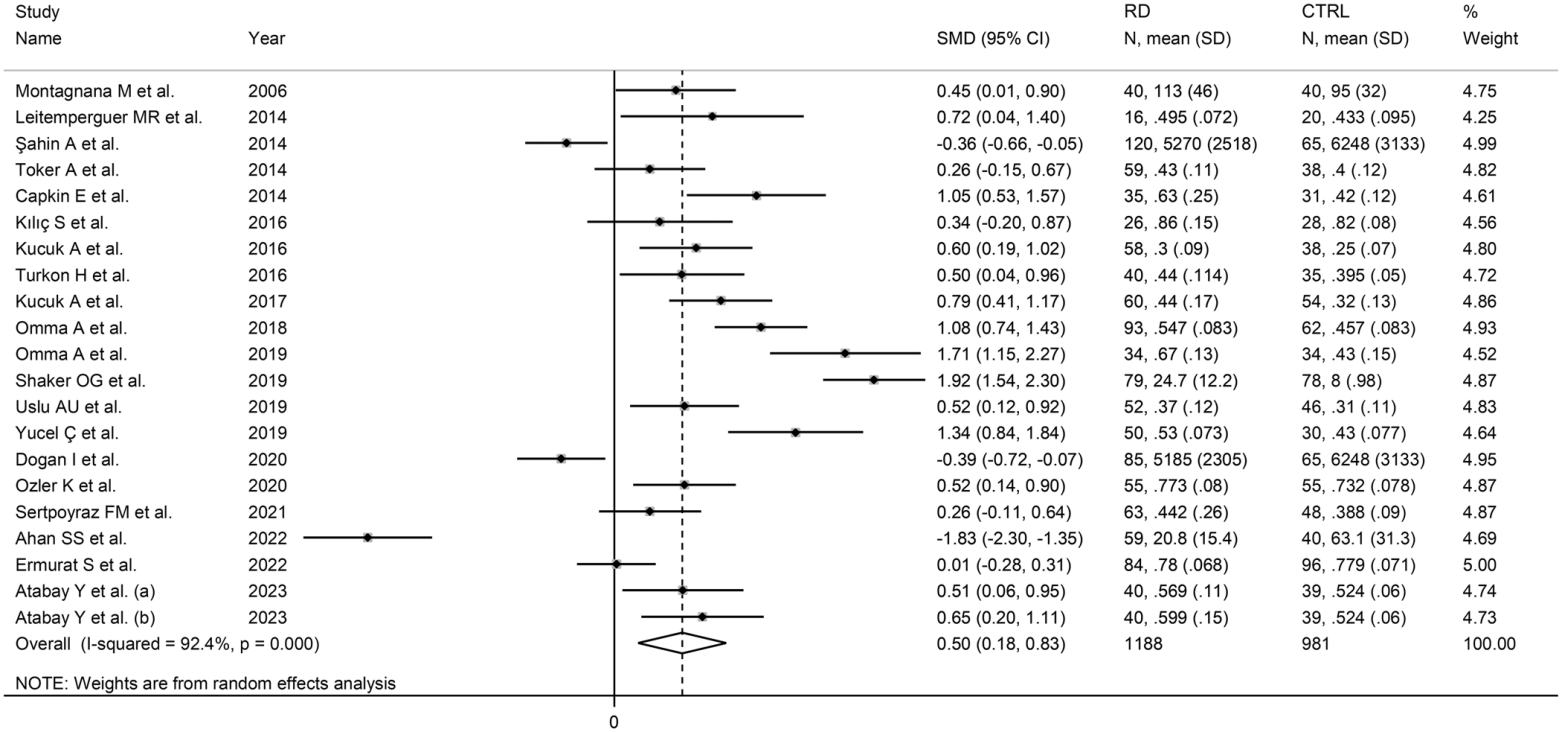
Forest plot of studies investigating ischemia‐modified albumin in patients with rheumatic diseases and healthy controls.

**Figure 3:**
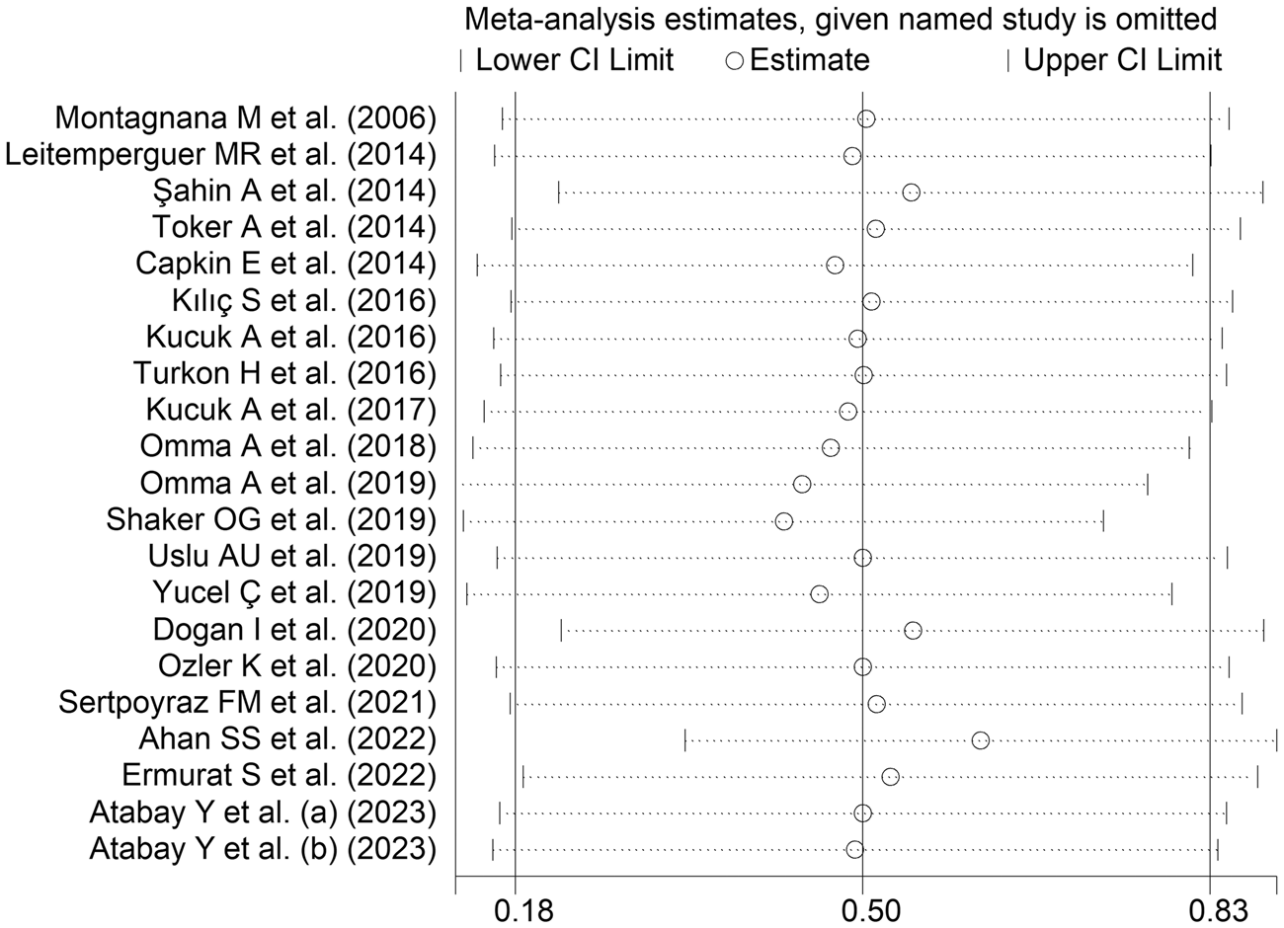
Sensitivity analysis of the association between ischemia‐modified albumin and rheumatic diseases.

The forest plot showed that IMA concentrations were significantly higher in RD patients taken as a whole when compared to controls (SMD = 0.50, 95% CI: 0.18−0.83, *p* = .003; *I* ^2^ = 92.4%, *p* < .001; Figure 2). Sensitivity analysis confirmed the stability of the results, with corresponding pooled SMD values ranging between 0.43 and 0.61 (Figure 3).

### 3.4 Publication bias

There was no significant publication bias according to either the Begg's (*p* = .17) or the Egger's (*p* = .21) test.

### 3.5 Meta‐regression and subgroup and analysis

**Figure 4:**
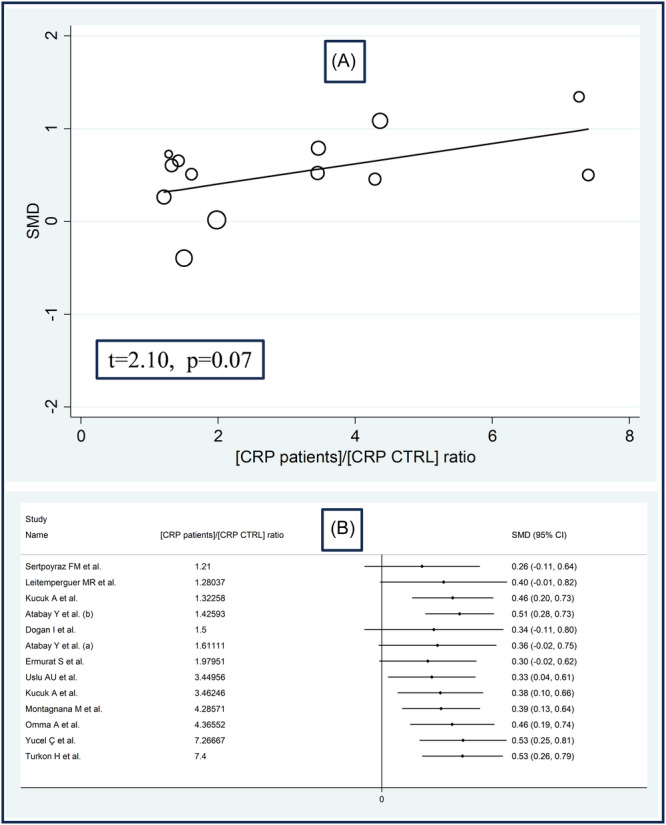
Bubble plot reporting the univariate meta‐regression analysis between the effect size and C‐reactive protein (CRP) (A) and cumulative meta‐analysis of ischemia‐modified albumin concentrations based on CRP concentrations (B).

No significant associations were observed in meta‐regression between the effect size and age (*t* = −1.23, *p* = .24), male to female ratio (*t* = 0.76, *p* = .46), publication year (*t* = −0.53, *p* = .60), sample size (*t* = −0.84, *p* = .41), BMI (*t* = 0.03, *p* = .98), ESR (*t* = 1.68, *p* = .13), or mean disease duration (*t* = 1.80, *p* = .11). There was a nonsignificant association trend with CRP (*t* = 2.10, *p* = .07; Figure 4A), also evident by cumulative analysis based on CRP values of RD patients performed using the metacum command (Figure 4B).

**Figure 5:**
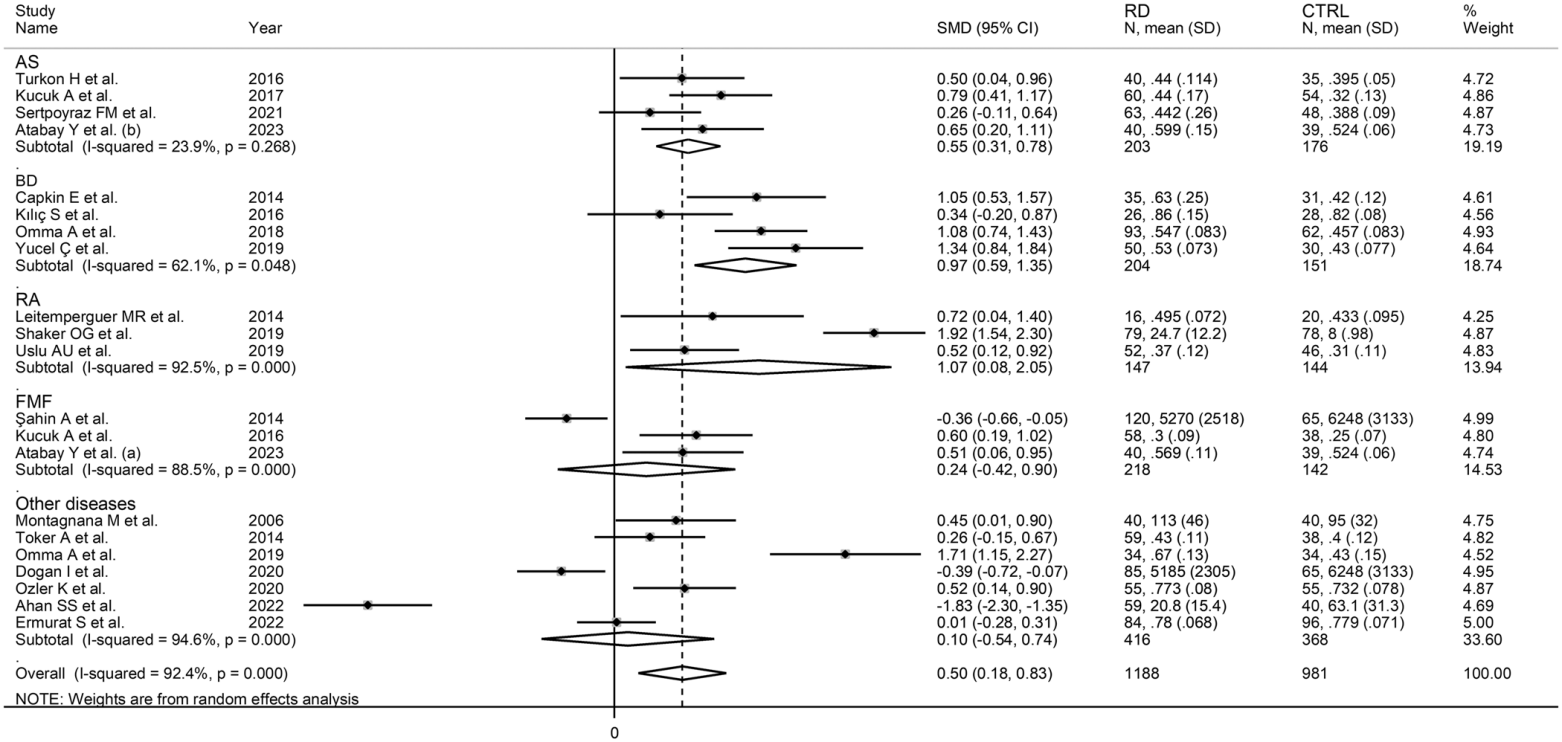
Forest plot of studies investigating ischemia‐modified albumin in patients with rheumatic diseases and healthy controls according to the type of rheumatic disease.

**Figure 6:**
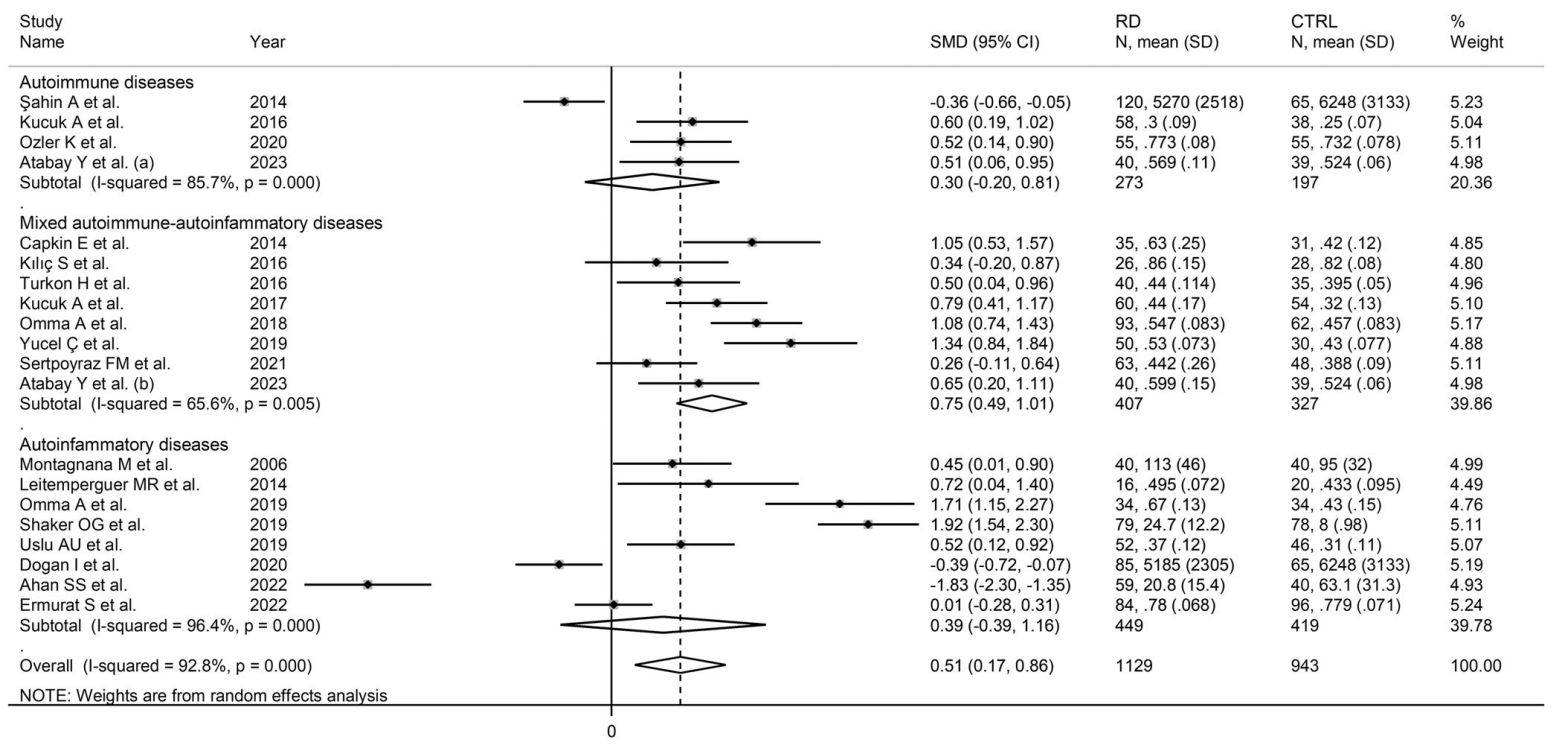
Forest plot of studies investigating ischemia‐modified albumin in patients with rheumatic diseases and healthy controls according to the presence of autoimmune, mixed autoimmune‐autoinflammatory, and autoinflammatory diseases.

**Figure 7:**
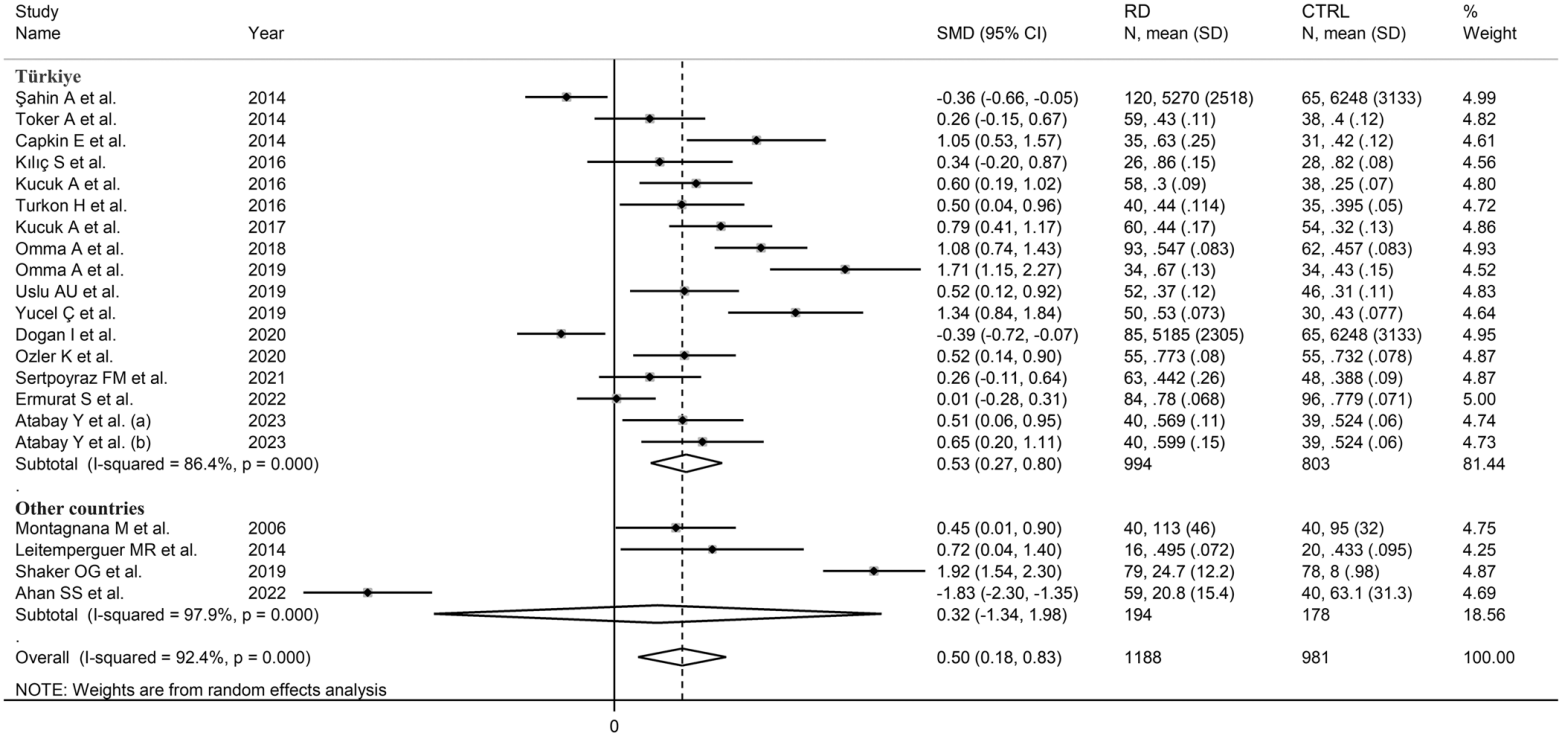
Forest plot of studies investigating ischemia‐modified albumin in patients with rheumatic diseases and healthy controls according to the country where the study was conducted.

**Figure 8:**
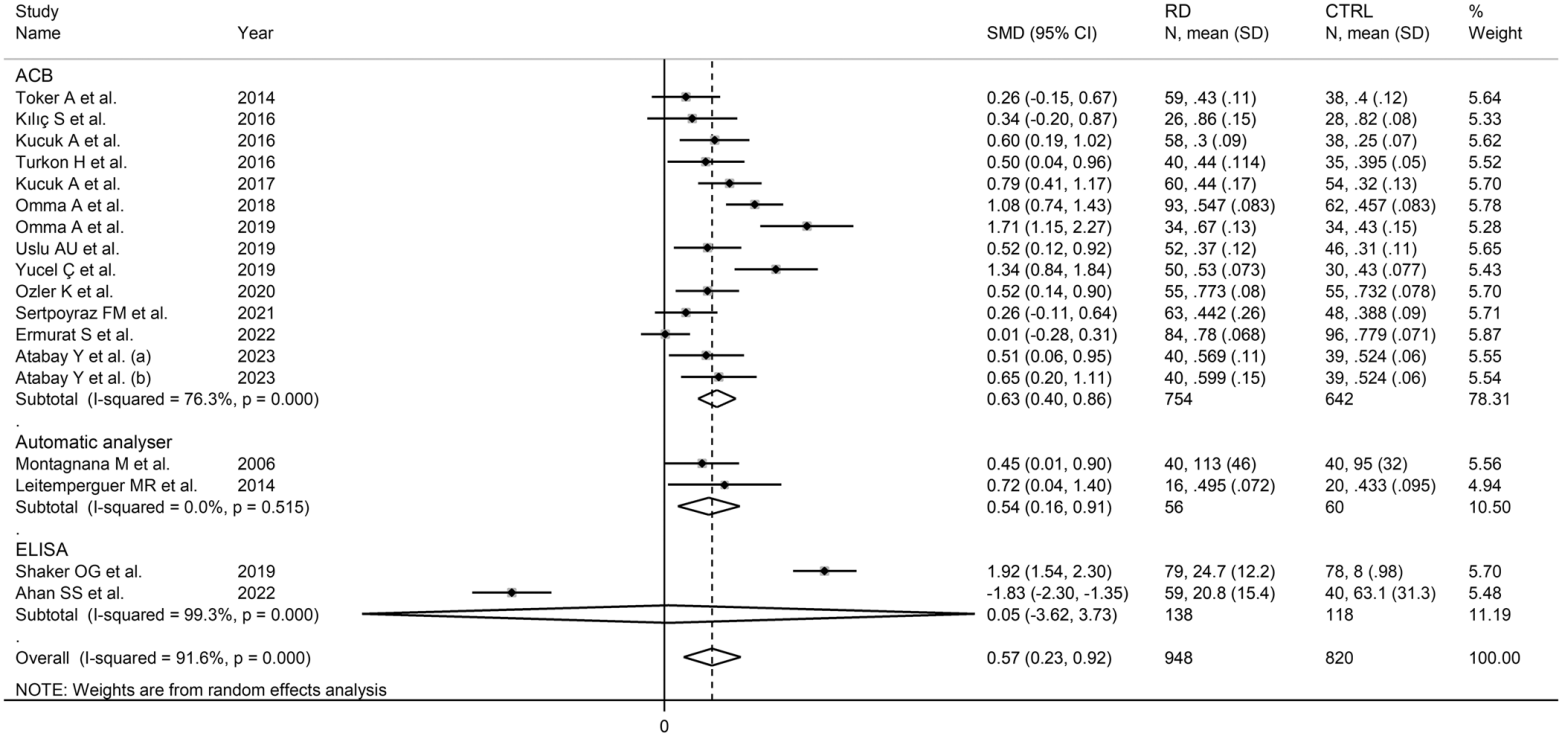
Forest plot of studies investigating ischemia‐modified albumin in patients with rheumatic diseases and healthy controls according to the analytical method used to measure ischemia‐modified albumin.

In subgroup analysis, the pooled SMD was significantly different in studies investigating AS (SMD = 0.55, 95% CI: 0.31−0.78, *p* < 0.001; *I* ^2^ = 23.9%, *p* = .27), BD (SMD = 0.97, 95% CI: 0.59−1.35, *p* < .001; *I* ^2^ = 62.1%, *p* = .048), and RA (SMD = 1.07, 95% CI: 0.08−2.05, *p* = .033; *I* ^2^ = 98.5%, *p* < 0.001), but not FMF (SMD = 0.24, 95% CI: −0.42 to 0.90, *p* = .48; *I* ^2^ = 88.5%, *p* < .001; Figure 5), with a relatively lower between‐study variance in the AS subgroup. The pooled SMD was significantly different in studies investigating mixed autoimmune‐autoinflammatory diseases (SMD = 0.75, 95% CI: 0.49−1.01, *p* < .001; *I* ^2^ = 65.6%, *p* = .005), but not autoimmune (SMD = 0.30, 95% CI: −0.20 to 0.81, *p* = .24; *I* ^2^ = 85.7%, *p* < .001), or autoinflammatory diseases (SMD = 0.39, 95% CI: −0.39 to 1.16, *p* = .33; *I* ^2^ = 96.4%, *p* < .001; Figure 6), with relatively lower between‐study variance in the mixed autoimmune‐autoinflammatory diseases subgroup. In addition, the pooled SMD was significantly different in studies conducted in Turkey (SMD = 0.53, 95% CI: 0.27−80, *p* < .001; *I* ^2^ = 86.4%, *p* < .001), but not in other countries (SMD = 0.32, 95% CI: −1.34 to 1.98, *p* = .70; *I* ^2^ = 97.9%, *p* < .001; Figure 7). Furthermore, the pooled SMD was significantly different in studies using the ACB assay (SMD = 0.63, 95% CI: 0.40−0.86, *p* < .001; *I* ^2^ = 76.3%, *p* < .001) and automatic analyzers (SMD = 0.54, 95% CI: 0.16−0.91, *p* = .005; *I* ^2^ = 0.0%, *p* = .515), but not ELISA (SMD = 0.05, 95% CI: −3.62 to 3.73, *p* = .98; *I* ^2^ = 99.3%, *p* < .001; Figure 8), with a virtually absent heterogeneity in the automatic analyzer subgroup.

### 3.6 Certainty of evidence

The overall level of certainty remained low (rating 2) after considering the low‐moderate risk of bias in virtually all studies (no change), the high but partially explainable heterogeneity (no change), the lack of indirectness (no change), the moderate effect size (SMD = 0.50, no change), and the absence of publication bias (no change).

## 4 DISCUSSION

In our systematic review and meta‐analysis, IMA concentrations were significantly higher in RD patients taken as a whole when compared to healthy controls. However, these alterations differed according to specific RDs and broad RD categories. Specifically, the between‐group differences in IMA concentrations versus controls were significant in patients with AS, BD, and RA but not in patients with FMF. Furthermore, they were significant in patients with mixed autoimmune‐autoinflammatory diseases but not in those with autoimmune or autoinflammatory diseases. In meta‐regression analysis, there were no significant associations between the effect size of the between‐group differences and various patient and study characteristics, particularly ESR, CRP, and mean RD duration. These observations suggest that the information provided by measuring IMA concentrations does not simply replicate that provided by conventional markers of inflammation and that the between‐group differences in IMA concentrations are also evident in patients with relatively short RD duration, supporting the potential clinical utility of IMA as a biomarker of early RDs. Further subgroup analyses highlighted the presence of significant differences in the pooled SMD in studies conducted in Turkey but not in other countries and the potential influence of the analytical method used in determining the between‐group differences in IMA concentrations.

Although there is ongoing uncertainty regarding the exact chemical reactions involved during the biotransformation of native albumin into IMA in the context of an ischemic event, the opposite biotransformation, with the regeneration of albumin, is common following ischemia.^38^, ^90^ In subgroup analysis, the pooled SMD was significantly different using the ACB assay and automatic analyzers, but not ELISA. These methods are generally characterized by high sensitivity and specificity however they are not yet available for routine clinical use.^38^ The majority of studies selected in our systematic review and meta‐analysis, 14 out of 20, used the ABC method.^91^ However, the results with this method may be influenced by pH fluctuations, denaturing agents, or specific pharmacological agents.^38^ Furthermore, IMA concentrations are commonly expressed as absorbance units, which may depend on operator experience and/or the sensitivity of the equipment, and internal standards have been obtained by some investigators in their laboratories.^38^ Although these factors might account for the high heterogeneity in our meta‐analysis it should also be emphasized that no consensus exists regarding the exact chemical reactions involved in the biotransformation of albumin into IMA.^38^ To address these issues, methods based on immunological reactions using antibodies to IMA are being investigated.^38^

The pathophysiological role of IMA has been primarily investigated in cerebrovascular disease and acute coronary syndrome.^41^, ^42^, ^43^, ^44^, ^45^, ^46^ However, significant alterations in IMA concentrations have also been reported in congestive heart failure,^47^ neurodegenerative disorders,^48^ pregnancy disorders,^92^ and cancer.^52^ These observations support the proposition that the increase in IMA concentrations observed in a wide range of conditions without a clinically overt ischemic process is primarily due to a pro‐oxidant state and, potentially, acidosis, rather than ischemia.^93^, ^94^, ^95^, ^96^, ^97^ Oxidative stress and, to a certain extent, local and/or systemic acidosis are also likely to account for the elevations in IMA concentrations observed in our study in RD patients.^53^, ^54^, ^55^, ^56^, ^57^, ^58^, ^59^, ^60^ Regardless of the exact biochemical and cellular mechanisms responsible for the increased IMA concentrations in RD patients, prospective studies should investigate whether IMA can enhance the diagnosis, monitoring, and management in this patient group before introducing its measurement in routine practice.

Our study has several strengths, including the assessment of IMA in patients with several types of RDs and broad RD classifications, the investigation of possible associations between the effect size of the between‐group differences in IMA concentrations, and several study and patient characteristics, particularly CRP, ESR, and RD duration, and a comprehensive assessment of the risk of bias and the certainty of evidence. Furthermore, the results of the meta‐analysis were stable in sensitivity analysis. However, important limitations should also be acknowledged, including the relatively limited number of RDs analyzed (SSc, RA, FMF, fibromyalgia, BD, AS, IgAV, SS, OA, AAV, and SLE) and the fact that the majority of studies, 16 out of 20, were conducted in Turkey, which curtails the generalizability of the results. This issue requires addressing in further research, also given that some evidence suggests the presence of significant differences in IMA concentrations between ethnic groups.^98^

## 5 CONCLUSIONS

Taken together, the results of our systematic review and meta‐analysis expand the current knowledge regarding the pathophysiological and diagnostic role of IMA, suggesting its potential utility as a biomarker of RDs, including patients with early forms of the disease. However, additional research is required to confirm these observations, to investigate the presence of similar alterations in patients with other types of RDs and different ethnicity, and to determine whether IMA can enhance diagnosis, monitoring, and management in prospective studies before its introduction in clinical practice.

## AUTHOR CONTRIBUTIONS

**Arduino A. Mangoni**: Conceptualization; methodology; validation; writing—original draft; writing—review and editing. **Angelo Zinellu**: Conceptualization; formal analysis; methodology; validation; writing—review and editing.

## CONFLICT OF INTEREST STATEMENT

The authors declare no conflict of interest.

## ETHICS STATEMENT

Ethics approval was not required as this was a systematic review of published studies. Patient consent was not required as this was a systematic review of published studies.

## Supporting information

Supporting information.

Supporting information.

## Data Availability

The data supporting the findings of this systematic review and meta‐analysis are available from A. Z. upon reasonable request.
